# Identification of both GABA_A_ receptors and voltage-activated Na^+^ channels as molecular targets of anticonvulsant α-asarone

**DOI:** 10.3389/fphar.2014.00040

**Published:** 2014-03-11

**Authors:** Ze-Jun Wang, Simon R. Levinson, Liqin Sun, Thomas Heinbockel

**Affiliations:** ^1^Department of Anatomy, College of Medicine, Howard UniversityWashington, DC, USA; ^2^Department of Physiology and Biophysics, University of Colorado Denver School of MedicineAurora, CO, USA

**Keywords:** GABA_A_ receptors, sodium channel blocker, α-asarone, central nerve Na_**v**_1.2 channel, olfactory bulb, anticonvulsant, sensitization of cough reflexes

## Abstract

Alpha (α)-asarone, a major effective component isolated from the Chinese medicinal herb *Acorus tatarinowii*, is clinically used as medication for treating epilepsy, cough, bronchitis, and asthma. In the present study, we demonstrated that α-asarone targets central nervous system GABA_A_ receptor as well as voltage-gated Na^+^ channels. Using whole-cell patch-clamp recording, α-asarone inhibited spontaneous firing of output neurons, mitral cells (MCs), in mouse olfactory bulb brain slice preparation and hyperpolarized the membrane potential of MCs. The inhibitory effect of α-asarone persisted in the presence of ionotropic glutamate receptor blockers but was eliminated after adding a GABA_A_ receptor blocker, suggesting that GABA_A_ receptors mediated the inhibition of MCs by α-asarone. This hypothesis was supported by the finding that α-asarone evoked an outward current, but did not influence inhibitory postsynaptic currents (IPSCs). In addition to inhibiting spontaneous firing, α-asarone also inhibited the Na_v_1.2 channel, a dominant rat brain Na^+^ channel subtype. The effects of α-asarone on a defined Na_v_1.2 were characterized using transfected cells that stably expressed the Na_v_1.2 channel isoform. α-Asarone displayed strong tonic inhibition of Na_v_1.2 currents in a concentration- and membrane potential-dependent fashion. α-Asarone reduced channel availability in steady-state inactivation protocols by enhancing or stabilizing Na^+^ channel inactivation. Both Na^+^ channel blockade and activation of GABA_A_ receptors provide a possible mechanism for the known anti-epileptic effects of α-asarone. It also suggests that α-asarone could benefit patients with cough possibly through inhibiting a Na^+^ channel subtype to inhibit peripheral and/or central sensitization of cough reflexes.

## INTRODUCTION

**FIGURE 1:**
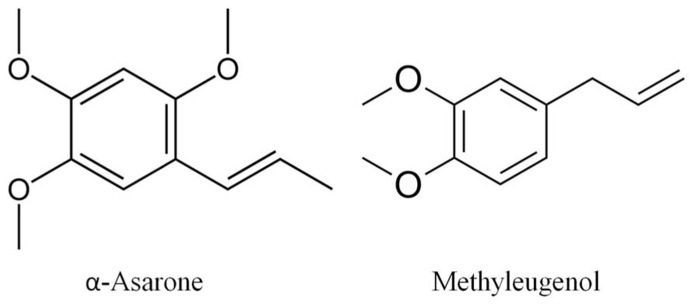
**Chemical structure of α-asarone and methyleugenol (4-allyl-1,2-dimethoxybenzene)**.

Alpha (α)-asarone (1-propenyl-2,4,5-methoxybenzol; **Figure 1**), a pharmacologically active component, is a natural product that originates in *Acorus tatarinowii* Schott, *A. calamus*, and in other plants (Dandiya and Menon, 1963; Hanson et al., 2005; Björnstad et al., 2009; Wang et al., 2012). *A. tatarinowii* is a Chinese herbal medicine which has been traditionally used for centuries to treat respiratory diseases and neuronal disorders such as epilepsy, cough, bronchitis, coma, and senile dementia (Chinese Pharmacopoeia Committee, 2010; Gu et al., 2010; Chen et al., 2013; Ni and Yu, 2013). The roots and rhizomes of *A. calamus* are traditionally used for many mental ailments (Dandiya and Menon, 1963) and for stomach cramps, dysentery, asthma, bronchitis, cough, anthelmintic, and as an insecticide (Mukherjee et al., 2007; Zuba and Byrska, 2012). In brief, these α-asarone containing herbs are heavily used for the treatment of neuronal disorders and respiratory diseases.

Several bioactivities of α-asarone have been reported recently, e.g., reducing the level of lipids (Cassani-Galindo et al., 2005), a neuroprotective effect (Limón et al., 2009) and reducing excitatory synaptic activity (Gu et al., 2010). In China, α-asarone is approved for the treatment of epilepsy, bronchitis, cough, and asthma, indicating that it is effective in treating two disorders, namely respiratory diseases and neuronal disorders in clinical practice. The beneficial effects of α-asarone on these two unrelated disorders could suggest that α-asarone binds to more than one protein target. The possible protein targets that α-asarone binds to and the molecular mechanism(s) underlying the distinct actions of α-asarone remain to be determined. It has been reported that α-asarone prolonged the falling down latent period of induced asthma and relaxed smooth muscle of trachea by antagonizing histamine and acetylcholine. Recently, it was reported that α-asarone alleviates epilepsy by modulating GABA_A_ receptors (Huang et al., 2013). Although potential mechanisms underlying the anti-epileptic effect and the beneficial effect for respiratory diseases have been studied, the pharmacological bases of these actions need to be further elucidated. Here, we hypothesize that α-asarone binds to distinct disease-specific protein targets to exert effects on both respiratory and neuronal disorders, respectively. Alternatively, it binds to distinct protein targets of one disorder and, thereby, displays synergistic effects for that disorder.

Cough is a prominent symptom of respiratory disorders such as chronic obstructive pulmonary disease, lung cancer, bronchitis, and asthma. Persistent cough may be due to peripheral and/or central sensitization of cough reflexes (Chung, 2002; Undem and Carr, 2010). Primary afferent nerves are regarded as novel target for antitussive therapy (Undem and Carr, 2010; Muroi et al., 2013; Spina and Page, 2013). Sodium channels play an important role in the cough reflexes. This has been shown by the fact that local anesthetics, blockers of sodium-dependent channels, are inhibitors of the cough reflex (Chung, 2002; Spina and Page, 2013). Recently, in an effort to functionally screen the bioactivity of a small group of natural products, we discovered that one of them, methyleugenol, emerged as a novel sodium (Na^+^) channel blocker (Wang et al., unpublished data). α-Asarone is a compound which is similar to methyleugenol in its chemical structure (**Figure 1**). Thus, we hypothesize that α-asarone may display similar functional properties such as Na^+^ channel blockade to inhibit the cough reflex.

α-Asarone has recently been shown to inhibit synaptically driven-spiking of hippocampal neurons to exert an anti-epileptic effect through activating GABA_A_ receptors (Huang et al., 2013). It is well known that many bioactive agents interact with more than one therapeutic target protein. For example, several anticonvulsant agents such as valproic acid, gabapentin, and topiramate have been shown to inhibit the function of Na^+^ channels and, at the same time, activate GABA_A_ receptors (Rho and Sankar, 1999). Felbamate and phenobarbital are anticonvulsant agents that interact with both *N*-methyl-D-aspartate (NMDA) and GABA_A_ receptors (Rho et al., 1994; McNamara, 1996). The pharmacological properties of these anticonvulsant agents suggest that an ideal anticonvulsant agent could target two or more epilepsy-related proteins or ion channels to control neural hyperexcitability. Thus, α-asarone may possibly interact with more than one therapeutic protein to exert its anti-epileptic effects. In central nervous system (CNS), voltage-gated type II/IIA Na^+^ channels (Na_v_1.2A) are preferentially localized in axons (Westenbroek et al., 1989). The tetrodotoxin (TTX)-sensitive channel isoform Na_v_1.2A has been studied as the target of multiple anti-epileptics and the inhibition of Na_v_1.2A is considered as an anticonvulsant mechanism (Ragsdale et al., 1991; Dupere et al., 1999). Therefore, based on the chemical similarity of α-asarone to methyleugenol, Na_v_1.2 is a good candidate to explore its possible regulation by α-asarone.

Epileptic seizures result from poorly controlled neuronal activity at a seizure focus and the subsequent spread of electrical excitation in brain circuits (Rall and Schleifer, 1990). It is not surprising that most effective anti-seizure medications inhibit neuronal excitability by modulating the function of several types of proteins such as Na^+^ channels, NMDA receptors, and GABA receptors (Rho and Sankar, 1999). Output neurons such as mitral cells (MCs) in the mouse main olfactory bulb (MOB) display their neuronal activity as spontaneous action potential firing, which can be modulated by intrinsic membrane receptors as well as synaptic inputs (Shepherd et al., 2004; Ennis et al., 2007; Wang et al., 2011). In the rodent MOB, MCs express high levels of different receptors such as GABA receptors (GABA_A_, GABA_B_), ionotropic and metabotropic glutamate receptors (NMDA, AMPA, metabotropic glutamate receptor 1 (mGluR1), kainite). Most of these receptor proteins are thought to be epilepsy-related (McNamara, 1996; Snell et al., 2000; Wang et al., 2002; Ennis et al., 2007). Therefore, compared to the synaptically driven-firing neurons (Huang et al., 2013), spontaneous firing MCs is a better choice for exploring the possible regulation of neuronal excitement by α-asarone. In this study, we used acute slices of the mouse MOB as well as stably transfected cells expressing the Na_v_1.2 channel α-subunit to determine the potential cellular targets of α-asarone.

## MATERIALS AND METHODS

### CELL CULTURE

The CNaIIA cell line (gift from Dr. W.A. Catterall) was derived from a Chinese hamster ovary (CHO-K1) cell line stably transfected with a cDNA encoding the rat brain type IIA Na^+^ channel (Na_v_1.2A; Ragsdale et al., 1991; Scheuer et al., 1992). CNaIIA cells were cultured in Roswell Park Memorial Institute medium (Gibco) with 5% fetal bovine serum, and 100 μg/ml streptomycin and 100 U/ml penicillin. G418 (400 μg/ml) was included to select for transfectants. Then the cells were passed and plated on glass coverslips in 35-mm dishes in a 5% CO_2_ atmosphere at 37°C for 1–3 days before experimentation.

### WHOLE-CELL VOLTAGE-CLAMP RECORDING FROM TRANSFECTED CHO CELLS

Na^+^ currents were recorded using the whole-cell patch clamp recording technique (Hamill et al., 1981). The cultured cells on coverslips were transferred to a handmade recording chamber and continuously perfused at room temperature with extracellular solution containing (in mM): 130 NaCl, 4 KCl, 1.5 CaCl_2_, 1.5 MgCl_2_, 5 glucose, 5 HEPES, 20 sucrose, pH 7.4 adjusted with NaOH. The recording chamber volume was approximately 0.4 ml and the flow rate was 0.6 ml/min. MP-285 micromanipulator (Sutter Instrument Co., Novato, CA, USA) was used to place the electrode onto the cell. Patch pipettes were pulled from borosilicate glass capillaries (Drummond Scientific Co., Broomall, PA, USA) on an electrode puller (Model P-97, Sutter Instrument Co.) and were filled with a 0.2 μm filtered internal solution containing (in mM): 90 CsF, 60 CsCl, 10 NaCl, 5 HEPES, pH 7.4 adjusted with NaOH. The pipettes had input resistance of 0.8–1.4 MΩ. Recordings were performed at room temperature (22°C) with a patch clamp EPC 9 (HEKA Elektronik GmbH, Germany) and were filtered at 5 kHz. Leakage currents were subtracted using a P/4 or P/2 protocol. Pulse (HEKA Elektronik) was used for experimental control and basic data analysis.

The methods of recording Na^+^ currents from Na_v_1.2 expressing cells were similar to our previous report (Wang et al., 2002). Cells with series resistance of less than 2.5 M were used for drug test experiments. Only cells with whole cell maximal Na^+^ currents of at least 1 nA were used in the analysis. Na^+^ currents recorded from Na_v_1.2A expressing cells always increased progressively within the first 20 min after establishing the whole-cell-recording configuration and then were relatively stable. Thus, drugs were applied only during this period (after 20 min), especially for testing tonic inhibition evoked by the drug. Time-dependent shifts (~0.5 mV before and after perfusion) in the inactivation curve of Na_v_1.2 have not been considered in calculating the shift by drug.

### SLICE PREPARATION

Wild type mice (C57BL/6J, Jackson Laboratory, Bar Harbor, ME, USA) were used in agreement with Institutional Animal Care and Use Committee and NIH guidelines. Juvenile (16- to 25-day old) mice were decapitated, and the MOBs were dissected out and immersed in artificial cerebrospinal fluid (ACSF, see below) at 4°C, as previously described (Heinbockel et al., 2004). Horizontal slices (400 μm-thick) were cut parallel to the long axis using a vibratome (Vibratome Series 1000, Ted Pella Inc., Redding, CA, USA). After 30 min at 30°C, slices were incubated in a holding bath at room temperature (22°C) until use. For recording, a brain slice was placed in a recording chamber mounted on a microscope stage and maintained at 30 ± 0.5°C by superfusion with oxygenated ACSF flowing at 2.5–3 ml/min.

### SLICE RECORDING AND DATA ACQUISITION

Visually guided recordings were obtained from cells in the MC layer with near-infrared differential interference contrast optics and a BX51WI microscope (Olympus Optical, Tokyo, Japan) equipped with a camera (C2400-07, Hamamatsu Photonics, Japan). Images were displayed on a Sony trinitron color video monitor (PVM-1353MD, Sony Corp. Japan). Recording pipettes (5–8 MΩ) were pulled on a Flaming-Brown P-97 puller (Sutter Instrument Co.) from 1.5 mm O.D. borosilicate glass with filament. Seal resistance was routinely >1 GΩ and liquid junction potential was 9–10 mV; reported measurements were not corrected for this potential. Data were obtained using a Multiclamp 700B amplifier (Molecular Devices, Sunnyvale, CA, USA). Signals were low-pass Bessel filtered at 2 kHz and digitized on computer disk (Clampex 10.1, Molecular Devices). Data were also collected through a Digidata 1440A Interface (Molecular Devices) and digitized at 10 kHz. Holding currents were generated under control of the Multiclamp 700B Commander.

The ACSF consisted of (in mM): NaCl 124, KCl 3, CaCl_2_ 2, MgSO_4_ 1.3, glucose 10, sucrose NaHCO_3_ 26, NaH_2_PO_4_ 1.25 (pH 7.4, 300 mOsm), saturated with 95 O_2_/5% CO_2_ (modified from Heyward et al., 2001). The standard pipette-filling solution consisted of (mM) K gluconate 125, MgCl_2_ 2, HEPES 10, Mg_2_ATP 2, Na_3_GTP 0.2, NaCl 1, EGTA 0.2. The ACSF consisted of the following (in mM): 124 NaCl, 3 KCl, 2 CaCl_2_, 1.3 MgSO_4_, 10 glucose, 4.4 sucrose, 26 NaHCO_3_, 1.25 NaH_2_PO_4_ (pH 7.4, 300 mOsm), saturated with 95% O_2_/5% CO_2_. For intracellular recording of spiking activity, the pipette-filling solution consisted of the following (in mM): 144 K-gluconate, 2 MgCl_2_, 10 HEPES, 5 Mg_2_ATP, 0.5 Na_3_GTP, 2 NaCl, 0.2 EGTA. Low-Cl^-^-based pipette solution contained the following (in mM): 125 cesium methanesulfonate (CsMeSO_3_), 1 NaCl, 10 phosphocreatine di–tris salt, 5 ATP, 0.5 GTP, 0.5 EGTA, 10 HEPES, 10 QX-314 [2(triethylamino)*-N-*(2,6-dimethylphenyl) bromide], pH 7.3 with 1 N CsOH (290 mOsm). High-Cl^-^-based pipette solution contained the following (in mM): 110 cesium chloride, 10 tetraethylammonium–Cl, 2 NaCl, 10 phosphocreatine di–tris salt, 5 ATP, 0.5 GTP, 0.5 EGTA, 10 HEPES, 10 QX-314, pH 7.3 with 1 N CsOH (290 mOsm).

### CHEMICALS AND DRUG APPLICATION

For all experiments, drugs were bath perfused at the final concentrations indicated by dissolving aliquots of stock solution in ACSF or in extracellular solution. α-Asarone was supplied by the National Institute for the Control of Pharmaceutical and Biological Products (Beijing, China). Stock solution of 40 mM α-asarone was prepared in dimethyl sulfoxide (DMSO) and then diluted to the desired concentrations for experiments (final concentration of DMSO in bath < 0.1%). The following drugs were also bath applied: L-2-amino-5-phosphonopentanoic acid (AP5, APV), 6-cyano-7-nitroquinoxaline-2-3-dione (CNQX), 2-(3-carboxypropyl)-3-amino-6-(4 methoxyphenyl)-pyridazinium bromide (gabazine, SR-95531). Chemicals were supplied by Tocris (Ellisville, MO). Control recordings showed that 0.1% DMSO had no detectable effects on MC activity and the Na^+^ currents in transfected CHO cells.

### DATA ANALYSIS

The data obtained with cultured CHO cells were analyzed using a combination of PulseFit (HEKA Elektronik) and SigmaPlot 9.0 (Jandel Scientific, Corte Madera, CA, USA) software. The data obtained with slice recordings were analyzed using a combination of Clampfit 10.2 (Molecular Devices) and Origin8 (OriginLab Corporation, Northampton, MA, USA). All results are presented as the mean SEM. Tests for statistical significance were performed using paired Student’s *t*-tests, and one-way ANOVA followed by the Bonferroni test for multiple comparisons.

## RESULTS

In culture preparations, 20–30% of Na_v_1.2**cells displayed fast, transient inward currents following depolarization. The maximal peak inward currents of Na_v_1.2 ranged from 0.7 to 8 nA. However, in a few cells, it went up to 20 nA. These currents were blocked completely by 0.5 μM TTX, confirming their identity as uncontaminated Na^+^ currents under these recording conditions. In slices, MCs were identified visually by their soma location and relatively large soma size, and by their input resistance (297 ± 19.2 MΩ, *n* = 46). Spontaneous firing is an intrinsic property of MCs (Ennis et al., 2007).

### α-ASARONE TONICALLY INHIBITED RAT BRAIN Na_**v**_1.2 CURRENTS

A specific Na^+^ channel subtype (Na_v_1.2) is known to be dominant in the rat brain (Westenbroek et al., 1989). To determine whether α-asarone directly interacted with central nervous Na^+^ channels, we subjected the drug to procedures similar to those used for phenytoin and carbamazepine (CBZ) characterization (Ragsdale et al., 1991).

**FIGURE 2:**
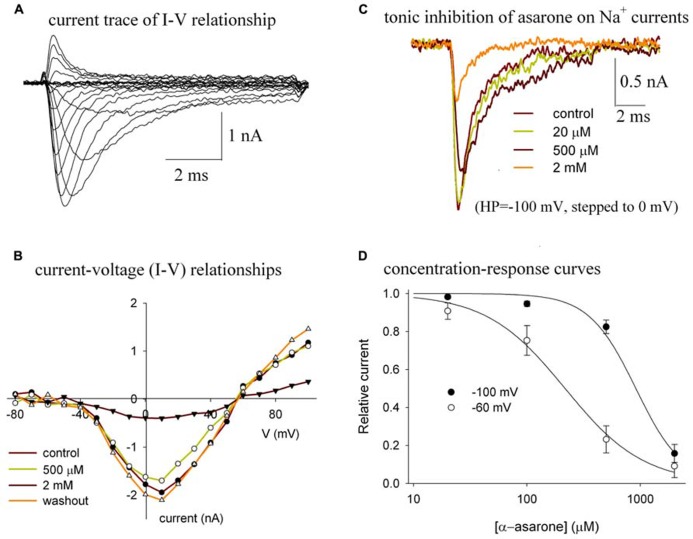
**(A–D)** Tonic inhibition of α-asarone on Na_**v**_1.2 channels. **(A) **The current traces of Na_v_1.2 illustrate the current–voltage (I–V) relationship of the channel. **(B) **The I–V relationships in control and in the presence of various concentrations of asarone. The currents were elicited by stepping to various depolarized potentials (ranging from – 80 to +100 mV in 10-mV increments) for 9 ms, and then returning to the holding potential of –100 mV. Peak currents at each depolarized potential were measured. The data are from a representative cell. **(C) **Superimposed current traces recorded before (control), during the application of 20 μM, 500 μM, and 2 mM α-asarone. The currents were recorded from the same cell and were elicited by a 10-ms pulse to 0 mV from the -100 mV holding potential. **(D) **Concentration–response curves for the inhibition of Na^+^ currents by α-asarone at different holding potentials. The cells were held at –100 mV, and –60 mV, respectively, and stepped to 0 mV for 10 ms. The peak currents in the presence of asarone were normalized to the corresponding control peak currents, and then averaged. Each point was the mean ± SEM of 4–6 cells. The lines are best fits for data to the Hill equation: y=1-x^n^/(K_d_^n^+x^n^), where *y *is the fractional current, *K*_d_ is the apparent dissociation constant for asarone, and *n *is the Hill coefficient. *K*_d_ and *n *were estimated using a Marquadt non-linear least-squares routine.

**FIGURE 3:**
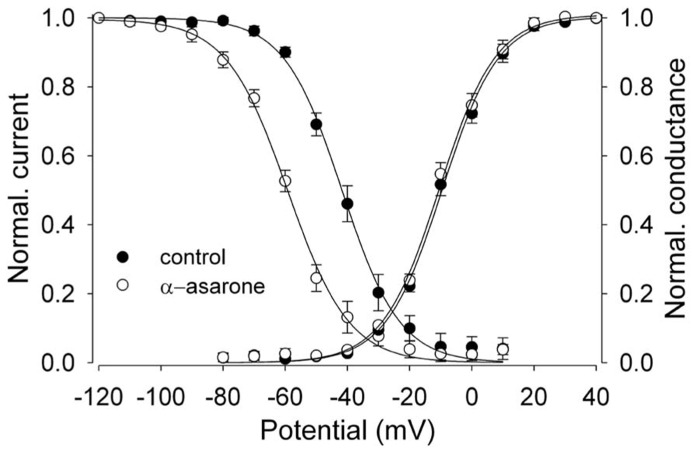
**α-asarone on voltage-dependent availability and activation of Na_v_1.2 channels**. The shift of inactivation curves of Na_v_1.2 channels by 500 μM asarone (*n* = 5). The voltage dependence of steady-state inactivation (*h*_∞_) was examined by applying 500-ms prepulse potentials from –120 to 10 mV in 10-mV increments from a holding potential of –100 mV before stepping to the test potential (0 mV) for 35 ms. The peak current (I) for each cell was normalized with respect to the first value measured at test potential (0 mV). *Conductance–voltage relationship:* from the peak Na^+^ currents obtained in **Figures 2A,B**, the Na^+^ conductance values (*G*) in the absence and presence of 500 μM α-asarone were calculated, normalized to the maximum in control, and plotted as a function of membrane potentials (*V*). The smooth curves through the data are drawn according to the equation: *y* = 1/1 - exp[(*V* - *V*_h_)/*k*]. Where *V* = membrane potential, *V*_h_**= the prepulse potential where the current is half-maximal, and *k* = the slope factor.

Bath application of α-asarone reversibly reduced the amplitude of the Na_v_1.2 peak currents (**Figure 2**). The currents of Na_v_1.2 shown in **Figure 2A** were elicited by stepping to various depolarized potentials from a holding potential of -100 mV. **Figure 2B** shows the current–voltage (I–V) relationship of Na_v_1.2 channels in control and in various concentrations of α-asarone, indicating that α-asarone inhibited Na^+^ currents. The shape of the I–V curve was unaffected by concentrations of α-asarone, suggesting that α-asarone, at the concentrations tested, had no effect on the voltage-dependent activation of Na^+^ channels, which was further analyzed in **Figure 3**. In **Figure 2C**, the currents were elicited by stepping to 0 mV from a holding potential of -100 mV, demonstrating that the tonic inhibition of Na_v_1.2 channels by α-asarone was concentration-dependent. **Figure 2D** shows the tonic inhibition by α-asarone on Na_v_1.2 currents which was determined by measuring concentration–response relationships at -100, and -60 mV holding potentials, respectively. At -100 mV, almost all channels were in the resting state which was observed by measuring the inactivation curve of Na_v_1.2 (shown in **Figure 3**). At -60 mV holding potential, about 15% of the channels were in the inactivated state. The averaged inhibition evoked by α-asarone at varying concentrations was well fit to the Hill equation. Based on a fitted Hill coefficient (*n*) value of 1.2 at a holding potential of -60 mV, it appeared that the stoichiometry of drug and receptor interaction was 1:1. The estimation of IC_50_**value obtained by fit to the Hill equation was 913 μM at -100 mV, and 217 μM at -60 mV, respectively. The result indicates that α-asarone exhibited a much higher affinity for channels at the -60 mV holding potential, where a subset of channels was in the inactivated state.

### α-ASARONE SHIFTED THE STEADY-STATE INACTIVATION CURVE

Na^+^ channel blockers like CBZ and phenytoin have voltage-dependent effects on the availability of Na^+^ currents, as seen by a shift of the steady-state inactivation curve to more negative potentials (Schwartz et al., 1989; Ragsdale et al., 1991). The steady-state inactivation (*h*_∞_) curve was examined to elucidate the effects of α-asarone on the voltage-dependent availability of Na_v_1.2 channels.

α-Asarone displayed voltage-dependent effects on the availability of Na_v_1.2 current. It shifted inactivation curves toward more negative potentials (**Figure 3**). The currents for each cell were normalized and averaged, and then fit to a single Boltzmann relationship from which the mean *V*_h_ and *k* values were calculated. The slope factors (*k*) of the curves were not affected at the concentrations of α-asarone we tested. The results suggest that α-asarone might interact preferentially with the inactivated state of the channels, which is typically found at depolarized potentials.

### α-ASARONE SUPPRESSED SPONTANEOUS SPIKING OF MITRAL CELLS AND HYPERPOLARIZED THEIR MEMBRANE POTENTIAL

The above results indicate that α-asarone acts on Na^+^ channels. In addition to Na^+^ channels, α-asarone may interact with other target proteins that are of clinical importance. MCs are output neurons in the MOB and spontaneously generate action potentials which can be modulated by intrinsic membrane properties as well as synaptic inputs (Shepherd et al., 2004; Ennis et al., 2007; Wang et al., 2011). We used the intrinsic properties of MCs such as spontaneous firing, membrane potential, and membrane conductance to evaluate the effect of α-asarone on neuronal activity and to determine the possible molecular mechanisms underlying the effects.

**FIGURE 4:**
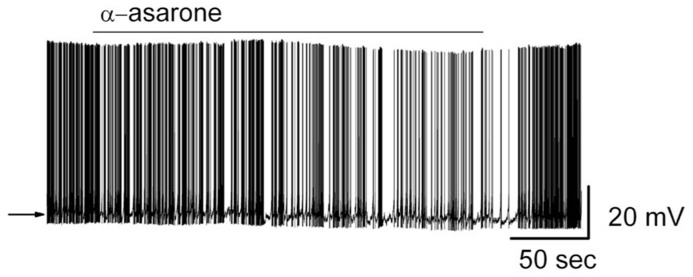
**α-asarone suppressed the spiking activity of MCs**. Original recording from a representative MC. Bath application of α-asarone reduced the firing rate and hyperpolarized the membrane potential. The arrow indicates the initial level of the resting potential.

Bath application of α-asarone modulated the spiking rate of MCs (**Figure 4**). Compared to control conditions, 20 μM α-asarone significantly reduced the MC firing rate. α-Asarone reversibly decreased MC firing rate from 4.7 ± 0.5 Hz to 2.9 ± 0.5 Hz (*n* = 9; *p *< 0.0001, paired* t-*test). The reduction of firing rate was accompanied by a hyperpolarization of the MC membrane potential from -50.8 ± 1.2 to -52.3 ± 1.2 mV (*n* = 8; *p *< 0.001, paired *t-*test). **Figure 4** illustrates the inhibitory effect of α-asarone in an original recording from a typical MC.

### GABA_**A**_ RECEPTORS RATHER THAN IONOTROPIC GLUTAMATE RECEPTORS WERE INVOLVED IN THE α-ASARONE-INDUCED INHIBITION OF NEURONAL EXCITABILITY

Ionotropic glutamate receptors play a critical role in the regulation of neuronal excitability in the MOB (Ennis et al., 2007). Blockade of ionotropic glutamate receptors can result in neuronal inhibition. We examined the effects of α-asarone-induced neuronal inhibition in the presence of AMPA/kainate and NMDA receptor blockers.

**FIGURE 5:**
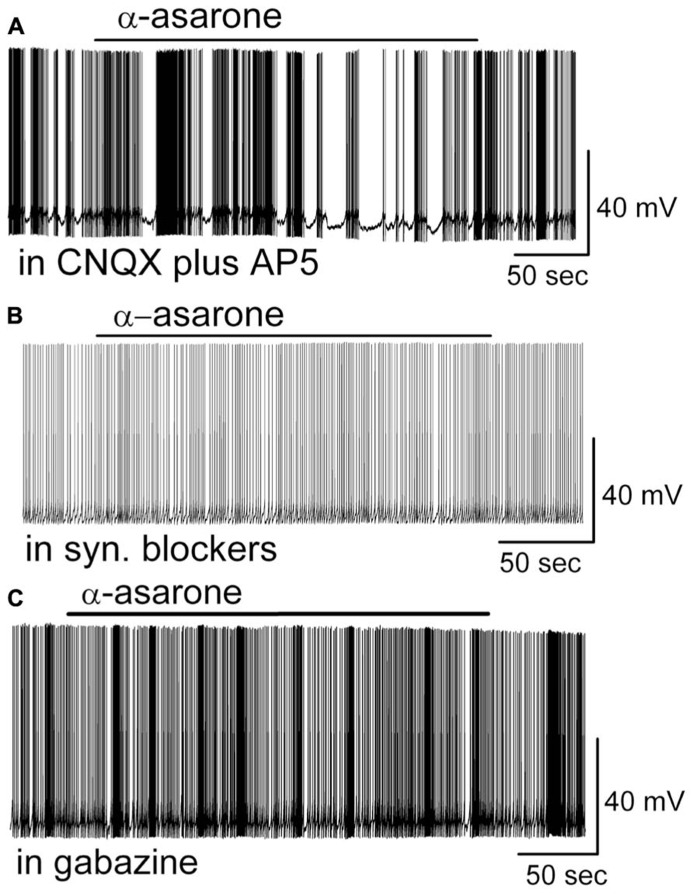
**(A–C)** A GABA_A_ receptor antagonist rather than AMPA/kainate or NMDA receptor antagonists blocked the α-asarone-induced inhibition of MCs. **(A)** Original recording obtained from a MC shows that α-asarone-induced suppression of neuronal firing recorded in a representative MC in the presence of iontropic glutamate receptor antagonists CNQX and D-AP5. **(B)** Current trace shows that α-asarone failed to evoke the inhibitory effects in the presence of fast synaptic blockers. **(C)** In gabazine, a GABA_A_ receptor antagonist, the firing rate remained the same. Current trace shows that gabazine eliminated the inhibitory effects of α-asarone.

In the presence of CNQX (10 μM, a potent AMPA/kainate receptor antagonist) and D-AP5 (50 μM, a potent NMDA receptor antagonist), the inhibitory effects of α-asarone persisted as seen by a reduction of the firing rate (in CNQX + D-AP5: 4.2 ± 0.6 Hz; in CNQX, D-AP5 plus α-asarone: 3.1 ± 0.5 Hz; *n* = 5; *p* < 0.01, paired *t-*test), and membrane hyperpolarization of MCs by -1.4 ± 0.2 mV (*n* = 5; *p* < 0.01, paired *t-*test; **Figure 5A**). In comparison with the results shown in **Figure 4**, these data indicate that CNQX plus D-AP5 had no any additional effect on α-asarone-induced suppression of MC activity (*p* > 0.05; ANOVA and Bonferroni *post hoc* analysis).

Even though the asarone-evoked inhibitory effects persisted in the presence of ionotropic glutamate receptor blockers, the inhibition was prevented by the application of synaptic blockers that contained both ionotropic glutamate receptor blockers and a GABA_A_ receptor blocker (synaptic blockers; CNQX, 10 μM; AP5, 50 μM; gabazine, 5 μM; **Figure 5B**). In synaptic blockers, α-asarone failed to reduce the firing rate of MC cells (in syn. blockers: 4.8 ± 0.9 Hz; in syn. blockers plus α-asarone: 4.8 ± 0.9 Hz; *n* = 5, *p* > 0.05, paired *t-*test), indicating GABA_A_ receptor may mediate the inhibitory effect of α-asarone.

To further examine the role of GABA_A_ receptors in α-asarone-evoked inhibition, the effect of α-asarone was measured during GABA_A_ receptor blockade. Blockade of GABA_A_ receptors can result in excitation or disinhibition of neurons. In the presence of gabazine, the inhibition of MC activity by α-asarone was eliminated (in gabazine: 5.3 ± 1.0 Hz; in gabazine plus α-asarone: 5.2 ± 1.0 Hz; *n* = 6, *p* > 0.05, paired *t-*test; **Figure 5C**). These results indicate that GABA_A_ receptors rather than ionotropic glutamate receptors were involved in the α-asarone-evoked inhibition of MC activity.

GABA_A_ receptors are present on MC dendrites in the MC layer of the MOB and play an important role in regulating MC excitability by suppressing neuronal activity (Laurie et al., 1992; Shepherd et al., 2004; Panzanelli et al., 2005). Bath application of γ-aminobutyric acid (GABA; 50 μM) dramatically decreased the firing rate of MCs (in control: 5.0 ± 0.8 Hz; in GABA: 1.7 ± 0.3 Hz; *n* = 6; *p *< 0.001, paired *t-*test) and hyperpolarized MCs by -1.1 ± 0.3 mV (*n* = 6; *p *< 0.05, paired *t-*test). The potent inhibition by GABA is consistent with previous reports showing that GABA receptors are abundant in MCs (Laurie et al., 1992; Persohn et al., 1992; Panzanelli et al., 2005).

**FIGURE 6:**
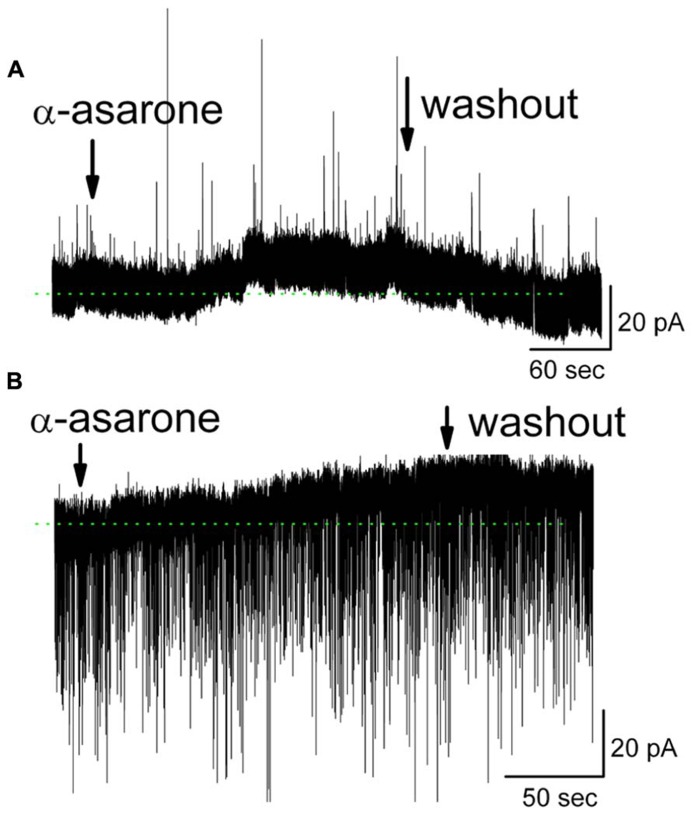
**α-Asarone induced outward currents in MCs**. **(A)** Original recording from a MC illustrates an outward current induced by 20 μM α-asarone. Low-Cl^-^ pipette solution containing sodium channel blocker QX-314 was used, and holding potential is 0 mV. **(B)** α-Asarone induced an outward current in MC with high-Cl^-^ pipette solution containing QX-314. Holding potential is at -60 mV. sIPSCs appear as downward deflections. The dotted green lines indicate the zero-current baselines.

Further evidence for the involvement of GABA_A_ receptors came from measurements of MC ionic currents induced by α-asarone. In whole-cell voltage clamp recording mode with Na^+^ channel blocker QX-314 included in the pipette solution, 20 μM α-asarone evoked an outward current in MCs of 11.8 ± 2.1 pA (*n* = 10; range 2.0–17 pA). The steady-state currents at the holding potential in α-asarone were measured and subtracted from that in ACSF (**Figure 6**). In the presence of gabazine, α-asarone-induced outward currents were blocked (-0.3 ± 1.5 pA, *n* = 4; the currents in α-asarone plus gabazine was subtracted from that in gabazine).

The gabazine-mediated blockade of α-asarone-evoked inhibition of MCs suggests that α-asarone either binds to GABA_A_ receptors to generate the inhibitory effects or it enhances extracellular GABA levels. α-Asarone-evoked outward currents may result from an enhancement of GABA release. This hypothesis was not supported by our observation that α-asarone was not able to increase the frequency of spontaneous inhibitory postsynaptic currents (sIPSCs) in MCs (**Figure 6**), suggesting that α-asarone enhanced tonic GABAergic inhibition rather than phasic GABAergic inhibition. With the recording conditions of **Figure 6A**, sIPSCs were upward and exhibited some rundown. Thus, a high-Cl^-^ pipette solution was used for sIPSCs recordings in subsequent experiments. In conditions of a high-Cl^-^ pipette solution (containing QX-314), the sIPSCs were downward deflected. α-Asarone induced an outward current as seen in **Figure 6B**, but did not change the frequency of sIPSCs (in control: 2.3 ± 0.3 Hz; in α-asarone: 2.4 ± 0.4 Hz) (*n* = 7; *p *> 0.05, paired *t-*test). The sIPSCs were completely eliminated by gabazine, indicating that sIPSCs were mediated by GABA acting on GABA_A_ receptors. In the presence of Na^+^ channel blocker (QX-314), α-asarone induced outward currents in MCs, but did not significantly alter sIPSC frequency, suggesting that asarone-evoked inhibition is mediated by direct action on GABA_A_ receptors rather than by indirect action through the elevation of tissue GABA levels.

## DISCUSSION

In the present study, we showed that α-asarone has a dual effect on neuronal proteins. It inhibited spontaneous activity of output neurons through activation of GABA_A_ receptors in the MOB and it inhibited voltage-gated Na^+^ currents of the rat brain Na^+^ channel α-subtype (Na_v_1.2). Our study shows that α-asarone markedly inhibited Na^+^ currents of Na_v_1.2, which are known to be dominant in the rat brain, in a concentration and membrane potential-dependent manner. α-Asarone reduced the availability of Na^+^ channels in steady-state inactivation protocols by enhancing or stabilizing Na^+^ channel inactivation. In addition to the blockade of Na^+^ channels, α-asarone inhibited neuronal activity of MCs. Ionotropic glutamate receptors were not involved in α-asarone-evoked inhibition of neuronal activity. Instead, α-asarone-evoked neuronal inhibition was mediated by direct activation of GABA_A_ receptors in the MOB rather than by increasing GABA release from GABAergic interneurons, suggesting the non-synaptic GABA_A_ receptors might be responsible for the effect of α-asarone inhibition. Therefore, we identified both GABA_A_ receptors and voltage-activated Na^+^ channels as cellular targets of α-asarone.

α-Asarone inhibits synaptically driven spiking in hippocampal neurons (Huang et al., 2013). Spiking in these hippocampal neurons is driven by glutamate and can be blocked by an AMPA receptor antagonist (Huang et al., 2013). The principal neurons in MOB are anatomically and functionally different from hippocampal neurons. MCs in the MOB are output neurons and exhibit their neuronal activity as spontaneous action potential firing, which can be modulated through intrinsic membrane properties as well as synaptic inputs (Shepherd et al., 2004; Ennis et al., 2007). Here, we found that α-asarone inhibited spontaneous, intrinsic spiking of MCs through activation of GABA_A_ receptors. Thus, α-asarone can inhibit both synaptically driven spiking and intrinsic spiking in mouse neurons, possibly to exert the anti-epileptic effects.

α-Asarone interacted directly with Na^+^ channels. Since Na^+^ channel blockers are often able to suppress sustained repetitive firing of neurons as use-dependent inhibition, we tested the effect of α-asarone on sustained repetitive firing of MCs in the presence of 5 μM gabazine (gabazine was used to block the action of α-asarone on GABA_A_ receptors). By injecting 100, 200 and 400 pA currents (500 ms) to depolarize MCs, the frequency of firing during the current injection was measured in the presence and absence of α-asarone. Unexpectedly, we did not see a significant reduction of firing rate by α-asarone (data not shown; only two out of 14 cells displayed the inhibition by 20 μM and 40 μM α-asarone on sustained repetitive firing). We are not sure why α-asarone did not show blockade of action potential in our recording condition. It is possible that the concentrations of α-asarone we used are not high enough to block firing. The failure of blocking firing at tested concentrations implies that Na channels may not contribute the a-asrarone-induced inhibition at the concentration of 20 and 40 μM. However, we do find use-dependent inhibition by 40 μM α-asarone in expressed CHO cell (data is not shown). Since α-asarone is a Na^+^ channel blocker, how can it fail to suppress sustained firing? Several scenarios might explain the lack of α-asarone effect on sustained firing. Firstly, increased input resistance (*R*_i_) are observed with GABA_A_ blockers (data not shown). The increased *R*_i_ by GABA_A_ blockers might partly explain the lack of effect of α-asarone on firing rate. Secondly, it is well known that a substantial proportion of patients with chronic epilepsy (up to 30%) remain refractory to antiepileptic drugs, i.e., not all neuronal activity at a seizure focus and the subsequent spread of electrical excitation in brain circuits can be well controlled by antiepileptic drugs including Na^+^ channels blockers. Possibly, Na^+^ channels blockers such as anticonvulsant CBZ may not be able to suppress epileptic-like activity in some specific area in the brain such as MOB. Thirdly, it has been reported that the action of the CBZ on Na^+^ channel appears to depend on the presence of the accessory β1 Na^+^ channel subunit (Kazen-Gillespie et al., 2000). The involvement of the β1 subunit in drug–Na^+^ channel interactions was shown by the fact that functional loss of accessory Na^+^ channel subunits is a feature of a number of neurological disorders, including epilepsy (Gastaldi et al., 1998; Ellerkmann et al., 2003; Patino et al., 2009; Uebachs et al., 2012). Na^+^ channel α- and β-subunit mRNAs exhibit differential expression in the CNS, suggesting that the subtypes have distinct physiological roles and contribute to the functional heterogeneity of neurons in the CNS (Whitaker et al., 2000, 2001). A localization study of Na^+^ channel β1 and β2 mRNA in the CNS demonstrates a differential expression in different neurons (Oh et al., 1994; Levy-Mozziconacci et al., 1998). It will be interesting to determine if accessory β1 or β2 subunits are functionally expressed in MCs. Fourthly, Na^+^ channel blockers such as eugenol have been reported to inhibit Na^+^ currents that are not dependent on the stimulus frequency in rat dorsal root ganglion neurons (Cho et al., 2008). It remains to be tested if commonly used Na^+^ channel blockers such as CBZ and phenytoin can effectively suppress sustained firing of MCs in the MOB. In the meantime, there is no report showing that anticonvulsants such as CBZ cause partial loss of olfaction. Since MCs are principal neurons in the MOB, the reduction/blockade of spontaneous firing of MCs may alter odor processing.

Persistent cough may be due to peripheral and/or central sensitization of cough reflexes (Chung, 2005; Undem and Carr, 2010). The primary afferent nerves are regarded as novel target for antitussive therapy (Undem and Carr, 2010; Muroi et al., 2013; Spina and Page, 2013). Lidocaine, a local anesthetic that acts by blocking Na^+^ channels, is an inhibitor of the cough reflex (Chung, 2002; Spina and Page, 2013). It is possible that α-asarone may exert its antitussive effect as a Na^+^ channel blocker to inhibit cough reflexes. Thus, our findings provide a possible mechanism underlying the effects of α-asarone on cough which is a prominent symptom of bronchitis, asthma, chronic obstructive pulmonary disease, and lung cancer. We identified both GABA_A_ receptors and voltage-activated Na^+^ channels as cellular targets of α-asarone. These cellular targets might be of therapeutic relevance in the treatment of both epilepsy and respiratory diseases. The precise mechanism by which α-asarone inhibits cough reflexes in animal models or humans remains to be elucidated.

## AUTHOR CONTRIBUTIONS

Ze-Jun Wang conducted the experiments, analyzed the data, provided intellectual input, and wrote the manuscript. Simon R. Levinson provided intellectual input. Liqin Sun conducted the experiments. Thomas Heinbockel provided intellectual input and wrote the manuscript.

## Conflict of Interest Statement

The authors declare that the research was conducted in the absence of any commercial or financial relationships that could be construed as a potential conflict of interest.

## References

[B1] Björnstad K. Helander A. Hultén P. Beck O. (2009). Bioanalytical investigation of asarone in connection with *Acorus calamus* oil intoxications. *J. Anal. Toxicol.* 33 604–609 10.1093/jat/33.9.604 20040135

[B2] Cassani-Galindo M. Madrigal-Bujaidar E. Chamorro G. Díaz F. Tamariz J. Espinosa-Aguirre J. J. (2005). In vitro genotoxic evaluation of three alpha-asarone analogues. *Toxicol. In Vitro* 19 547–552 10.1016/j.tiv.2005.01.007 15826813

[B3] Chen Q. X. Miao J. K. Li C. Li X. W. Wu X. M. Zhang X. P. (2013). Anticonvulsant activity of acute and chronic treatment with a-asarone from *Acorus gramineus* in seizure models. *Biol. Pharm. Bull.* 36 23–30 10.1248/bpb.b12-00376 23075695

[B4] Chinese Pharmacopoeia Committee (2010). *Pharmacopoeia of the People’s Republic of China*, Vol. 1. Beijing: Chemical Industry Press

[B5] Cho J. S. Kim T. H. Lim J. M. Song J. H. (2008). Effects of eugenol on Na^+^ currents in rat dorsal root ganglion neurons. *Brain Res.* 1234 53–62 10.1016/j.brainres.2008.09.030 18824159

[B6] Chung K. F. (2002). Cough: potential pharmacological developments. *Expert Opin. Invest. Drugs* 11 955–963 10.1517/13543784.11.7.955 12084006

[B7] Chung K. F. (2005). Drugs to suppress cough. *Expert Opin. Invest. Drugs* 14 19–27 10.1517/13543784.14.1.19 15709918

[B8] Dandiya P. C. Menon M. K. (1963). Effects of asarone and beta-asarone on conditioned responses, fighting behavior and convulsions. *Br. J. Pharmacol. Chemother.* 20 436–442 10.1111/j.1476-5381.1963.tb01480.x 14024874 PMC1703817

[B9] Dupere J. R. B. Dale T. J. Starkey S. J. Xie X. (1999). The anticonvulsant BW534U87 depresses epileptiform activity in rat hippocampal slices by an adenosine-dependent mechanism and through inhibition of voltage-gated Na^+^ channels. *Br. J. Pharmacol.* 128 1011–1020 10.1038/sj.bjp.0702881 10556938 PMC1571724

[B10] Ellerkmann R. K. Remy S. Chen J. Sochivko D. Elger C. E. Urban B. W. (2003). Molecular and functional changes in voltage-dependent Na^(^^+^^)^ channels following pilocarpine-induced status epilepticus in rat dentate granule cells. *Neuroscience* 119 323–333 10.1016/S0306-4522(03)00168-4 12770549

[B11] Ennis M. Hamilton K. A. Hayar A. (2007). “Neurochemistry of the main olfactory system,” in *Handbook of Neurochemistry and Molecular Neurobiology* ed. Lajtha A. *Sensory Neurochemistry*, 3rd Edn, Vol. 20 ed. Johnson D. A. (Heidelberg: Springer) 137–204

[B12] Gastaldi M. Robaglia-Schlupp A. Massacrier A. Planells R. Cau P. (1998). mRNA coding for voltage-gated sodium channel beta2 subunit in rat central nervous system: cellular distribution and changes following kainate-induced seizures. *Neurosci. Lett.* 249 53–56 10.1016/S0304-3940(98)00394-2 9672387

[B13] Gu Q. Du H. Ma C. Fotis H. Wu B. Huang C. (2010). Effects of alpha-asarone on the glutamate transporter EAAC1 in *Xenopus* oocytes. *Planta Med.* 76 595–598 10.1055/s-0029-1240613 19937551

[B14] Hamill O. P. Marty A. Neher E. Sakmann B. Sigworth F. J. (1981). Improved patch-clamp techniques for high-resolution current recording from cells and cell-free membrane patches. *Pfluegers Arch.* 391 85–100 10.1007/BF00656997 6270629

[B15] Hanson K. M. Gayton-Ely M. Holland L. A. Zehr P. S Söderberg B. C. (2005). Rapid assessment of beta-asarone content of *Acorus calamus* by micellar electrokinetic capillary chromatography. *Electrophoresis* 26 943–946 10.1002/elps.200410165 15714542

[B16] Heinbockel T. Heyward P. Conquet F. Ennis M. (2004). Regulation of main olfactory bulb mitral cell excitability by metabotropic glutamate receptor mGluR1. *J. Neurophysiol.* 92 3085–3096 10.1152/jn.00349.2004 15212418

[B17] Heyward P. Ennis M. Keller A. Shipley M. T. (2001). Membrane bistability inolfactory bulb mitral cells. *J. Neurosci.* 21 5311–5320 11438607 10.1523/JNEUROSCI.21-14-05311.2001 PMC6762867

[B18] Huang C. Li W. G. Zhang X. B. Wang L. Xu T. L. Wu D. (2013). Alpha-asarone from *Acorus gramineus* alleviates epilepsy by modulating A-type GABA receptors. *Neuropharmacology* 65 1–11 10.1016/j.neuropharm.2012.09.001 22975146

[B19] Kazen-Gillespie K. A. Ragsdale D. S. D’Andrea M. R. Mattei L. N. Rogers K. E. Isom L. L. (2000). Cloning, localization, and functional expression of sodium channel beta1A subunits. *J. Biol. Chem.* 275 1079–1088 10.1074/jbc.275.2.1079 10625649

[B20] Laurie D. J. Seeburg P. H. Wisden W. (1992). The distribution of 13 GABAA receptor subunit mRNAs in the rat brain. II. Olfactory bulb and cerebellum. *J. Neurosci.* 12 1063–1076 10.1523/JNEUROSCI.12-03-01063.1992 PMC6576040 1312132

[B21] Levy-Mozziconacci A. Alcaraz G. Giraud P. Boudier J. A. Caillol G. Couraud F. (1998). Expression of the mRNA for the beta 2 subunit of the voltage-dependent sodium channel in rat CNS. *Eur. J. Neurosci.* 10 2757–2767 10.1046/j.1460-9568.1998.00283.x 9758146

[B22] Limón I. D. Mendieta L. Díaz A. Chamorro G. Espinosa B. Zenteno E. (2009). Neuroprotective effect of alpha-asarone on spatial memory and nitric oxide levels in rats injected with amyloid-beta((25–35)). *Neurosci. Lett.* 453 98–103 10.1016/j.neulet.2009.02.011 19356601

[B23] McNamara J. O. (1996). “Drugs effective in the therapy of the epilepsies,” in *The Pharmacological Basis of Therapeutics* eds Hardman J. G. Limbird L. E. Molinoff P. B. Ruddon R. W. Gilman A. G. (New York: McGraw–Hill) 461–486

[B24] Mukherjee P. K. Kumar V. Mal M. Houghton P. J. (2007). In vitro cetylcholinesterase inhibitory activity of the essential oil from *Acorus calamus* and its main constituents. *Planta Med.* 73 283–285 10.1055/s-2007-967114 17286241

[B25] Muroi Y. Ru F. Chou Y. L. Carr M. J. Undem B. J. Canning B. J. (2013). Selective inhibition of vagal afferent nerve pathways regulating cough using Nav 1.7 shRNA silencing in guinea pig nodose ganglia. * Am. J. Physiol. Regul. Integr. Comp. Physiol.* 304 R1017–R1023 10.1152/ajpregu.00028.2013 23576611 PMC3680757

[B26] Ni G. Yu D. Q. (2013). Chemical constituents from rhizomes of *Acorus tatarinowii*. *Zhongguo Zhong Yao Za Zhi* 38 569–573 23713285

[B27] Oh Y. Sashihara S. Waxman S. G. (1994). In situ hybridization localization of the Na^+^ channel beta 1 subunit mRNA in rat CNS neurons. *Neurosci. Lett.* 176 119–122 10.1016/0304-3940(94)90885-0 7970226

[B28] Panzanelli P. Perazzini A. Z. Fritschy J. M Sassoè-Pognetto M. (2005). Heterogeneity of gamma-aminobutyric acid type A receptors in mitral and tufted cells of the rat main olfactory bulb. *J. Comp. Neurol.* 484 121–131 10.1002/cne.20440 15717305

[B29] Patino G. A. Claes L. R. Lopez-Santiago L. F. Slat E. A. Dondeti R. S. Chen C. (2009). A functional null mutation of SCN1B in a patient with Dravet syndrome. *J. Neurosci.* 29 10764–10778 10.1523/JNEUROSCI.2475-09.2009 19710327 PMC2749953

[B30] Persohn E. Malherbe P. Richards J. G. (1992). Comparative molecular neuroanatomy of cloned GABAA receptor subunits in the rat CNS. *J. Comp. Neurol.* 326 193–216 10.1002/cne.903260204 1336019

[B31] Ragsdale D. S. Scheuer T. Catterall W. A. (1991). Frequency and voltage-dependent inhibition of type IIA Na^+^ channels, expressed in a mammalian cell line, by local anesthetic, antiarrhythmic, and anticonvulsant drugs. *Mol. Pharmacol.* 40 756–765 1658608

[B32] Rall T. W. Schleifer L. S. (1990). “Drugs effective in the therapy of the epilepsies,” in *The Pharmacological Basis of Therapeutics* eds Goodman A. G. Rall T. W. Nies A. S. Taylor P. (New York: Pergamon Press) 436–462

[B33] Rho J. M. Donevan S. D. Rogawski M. A. (1994). Mechanism of action of the anticonvulsant felbamate: opposing effects on *N*-methyl-D-aspartate and GABAA receptors. *Ann. Neurol.* 36 677–678 10.1002/ana.410360424 8109904

[B34] Rho J. M. Sankar R. (1999). The pharmacologic basis of antiepileptic drug action. *Epilepsia* 40 1471–1483 10.1111/j.1528-1157.1999.tb02029.x 10565572

[B35] Scheuer T. West K. W. Maechler L. Catterall W. A. (1992). Efficient expression of rat brain Na^+^ channel type IIA α subunits in Chinese hamster ovary cells. *Neuron* 8 59–70 10.1016/0896-6273(92)90108-P 1309650

[B36] Shepherd G. W. Chen W. R. Greer C. A. (2004). “Olfactory bulb,” in *The Synaptic Organization of the Brain*, ed. G. M. Shepherd (New York: Oxford) 165–216

[B37] Snell L. D. Claffey D. J. Ruth J. A. Valenzuela C. F. Cardoso R. Wang Z. (2000). Novel structure having antagonist actions at both the glycine site of the *N*-methyl-D-aspartate receptor and neuronal voltage-sensitive sodium channels: biochemical, electrophysiological, and behavioral characterization. *J. Pharmacol. Exp. Ther.* 292 215–227 10604951

[B38] Spina D. Page C. P. (2013). Regulating cough through modulation of sensory nerve function in the airways. *Pulm. Pharmacol. Ther.* 26 486–490 10.1016/j.pupt.2013.03.011 23524012

[B39] Uebachs M. Albus C. Opitz T. Isom L. Niespodziany I. Wolff C. (2012). Loss of β1 accessory Na^+^ channel subunits causes failure of carbamazepine, but not of lacosamide, in blocking high-frequency firing via differential effects on persistent Na^+^ currents. *Epilepsia* 53 1959–1967 10.1111/j.1528-1167.2012.03675.x 23016711

[B40] Undem B. J. Carr M. J. (2010). Targeting primary afferent nerves for novel antitussive therapy. *Chest* 137 177–184 10.1378/chest.09-1960 20051402

[B41] Wang Y. Li F. Yang F. Q. Zuo H. L. Xia Z. N. (2012). Simultaneous determination of α-, β- and γ-asarone in *Acorus tatarinowii* by microemulsion electrokinetic chromatography with [BMIM]PF6 as oil phase. *Talant* 101 510–515 10.1016/j.talanta.2012.10.015 23158356

[B42] Wang Z. J. Snell L. D. Tabakoff B. Levinson S. R. (2002). Inhibition of neuronal Na^+^ channels by the novel antiepileptic compound DCUKA: identification of the diphenylureido moiety as an inactivation modifier. *Exp. Neurol.* 178 129–138 10.1006/exnr.2002.8029 12460615

[B43] Wang Z. J. Sun L. Jackson P. L. Scott K. R. Heinbockel T. (2011). A substituted anilino enaminone acts as a novel positive allosteric modulator of GABAA receptors in the mouse brain. *J. Pharmacol. Exp. Ther.* 336 916–924 10.1124/jpet.110.173740 21163867 PMC3061544

[B44] Westenbroek R. E. Merrick D. K. Catterall W. A. (1989). Differential subcellular localization of the RI and RII Na^+^ channel subtypes in central neurons. *Neuron* 3 695–704 10.1016/0896-6273(89)90238-9 2561976

[B45] Whitaker W. R. Clare J. J. Powell A. J. Chen Y. H. Faull R. L. Emson P. C. (2000). Distribution of voltage-gated sodium channel α-subunit and β-subunit mRNAs in human hippocampal formation, cortex, and cerebellum. *J. Comp. Neurol.* 422 123–139 10.1002/(SICI)1096-9861(20000619)422:1<123::AID-CNE8>3.0.CO;2-X 10842222

[B46] Whitaker W. R. Faull R. L. Dragunow M. Mee E. W. Emson P. C. Clare J. J. (2001). Changes in the mRNAs encoding voltage-gated sodium channel types II and III in human epileptic hippocampus. *Neuroscience* 106 275–285 10.1016/S0306-4522(01)00212-3 11566500

[B47] Zuba D. Byrska B. (2012). Alpha- and beta-asarone in herbal medicinal products. A case study. *Forensic Sci. Int.* 223 e5–e9 10.1016/j.forsciint.2012.08.015 22964166

